# Gallic and ascorbic acids supplementation alleviate cognitive deficits and neuropathological damage exerted by cadmium chloride in Wistar rats

**DOI:** 10.1038/s41598-022-18432-0

**Published:** 2022-08-24

**Authors:** Olamide Adebiyi, Kabirat Adigun, Praise David-Odewumi, Uthman Akindele, Funsho Olayemi

**Affiliations:** Department of Veterinary Physiology and Biochemistry, University of Ibadan, Ibadan, Nigeria

**Keywords:** Neuroscience, Physiology, Anatomy

## Abstract

Cadmium is a highly neurotoxic heavy metal that interferes with DNA repair mechanisms via generation of reactive oxygen species. The potentials of polyphenols and antioxidants as effective protective agents following heavy metal-induced neurotoxicity are emerging. We therefore explored the neuroprotective potentials of gallic and ascorbic acids in CdCl_2_-induced neurotoxicity. Seventy-two Wistar rats were divided into six groups. Group A received distilled water, B: 3 mg/kg CdCl_2_, C: 3 mg/kg CdCl_2_ + 20 mg/kg gallic acid (GA), D: 3 mg/kg CdCl_2_ + 10 mg/kg ascorbic acid (AA), E: 20 mg/kg GA and F: 10 mg/kg AA orally for 21 days. Depression, anxiety, locomotion, learning and memory were assessed using a battery of tests. Neuronal structure and myelin expression were assessed with histological staining and immunofluorescence. The Morris Water Maze test revealed significant increase in escape latency in CdCl_2_ group relative to rats concurrently treated with GA or AA. Similarly, time spent in the target quadrant was reduced significantly in CdCl_2_ group relative to other groups. Concomitant administration of gallic acid led to significant reduction in the durations of immobility and freezing that were elevated in CdCl_2_ group during forced swim and open field tests respectively. Furthermore, GA and AA restored myelin integrity and neuronal loss observed in the CdCl_2_ group. We conclude that gallic and ascorbic acids enhance learning and memory, decrease anxiety and depressive-like behavior in CdCl_2_-induced neurotoxicity with accompanying myelin-protective ability.

## Introduction

Naturally occurring elements with atomic weight and density at least 5 times greater than that of water have been described as heavy metals^1^. Globally, heavy metal toxicity is a major health threat affecting plants, animals and humans^2^. Cadmium is a heavy metal that currently has no known physiological function and poses health risks for animals and humans^3,4^. It is carcinogenic and ranked seventh most toxic occupational and environmental heavy metal^5,6^. Aside from its use as alloys in electroplating and manufacturing of pigments, it is also combined with nickel to make batteries and a by-product during the production of other heavy metals such as lead, zinc and copper^1,7,8^.

Cadmium exposure in humans usually occurs as a result of ingestion of contaminated food and water, cigarette smoking and cigarette smoke inhalation^2,4^. Its rate of excretion in the body is extremely low causing it to accumulate in soft tissues leading to neurotoxicity, nephrotoxicity, hepatotoxicity, endocrine and reproductive toxicities^9,10^. Exposure can lead to acute or chronic intoxications^2^ causing reactive oxygen species generation, initiation of apoptosis and interferences with DNA repair^7^.

Cadmium-induced neurotoxicity has been documented to result in decreased equilibrium, vasomotor dysfunction, peripheral neuropathy, learning and memory deficits^11^. Cadmium neurotoxicity has also been associated with onset of several neurodegenerative diseases such as Parkinson’s and Alzheimer’s that are characterized by alteration in the permeability of the blood–brain barrier^12^.

Gallic acid (3, 4, 5-trihydroxybenzoic acid) is one of the most abundant phenolic acids present in various plants. Its derivatives can be found in various fruits such as lemon, bananas, pineapples and some plants^13,14^. It is known to have antioxidant, anti-inflammatory, antimicrobial, anticancer, gastroprotective, cardioprotective and neuroprotective properties^15^. It exerts its neuroprotective effects by preventing the formation of free radicals, inhibiting the production of cytokines and maintenance of calcium homeostasis thereby helping to maintain neuronal plasticity^16^. It has been reported to improve cognitive and motor impairments in Wistar rats^16,17^.

Similarly, ascorbic acid is an antioxidant that protects the body from various adverse effects of free radicals and toxins^18^. Furthermore, it has been documented to decrease oxidative stress and formation of protein aggregates in neurodegenerative disorders^19^. Despite several studies reporting the protective effects of these two antioxidants in Cd-induced nephrotoxicity, hepatotoxicity and reproductive toxicity in animals^20–22^, to the best of our knowledge, their roles in the protection of Cd-induced behavioral alterations and neuropathology are yet to be assessed. In this study, we therefore evaluated the neuro-protective effects of gallic and ascorbic acids against cadmium-induced neurotoxicity and behavioral alterations in rodent model.

## Materials and methods

### Experimental animals

A total of seventy-two Wistar rats (150–180 g), aged 8–10 weeks of both sexes were used for this study. The rats were housed under standard housing condition, fed regular pelletized diet and water ad libitum under a 12/12 h light/dark cycle.

### Chemicals

Gallic acid (#20110905, Tianjin Kermel Chemical Reagents Limited, China), ascorbic acid (#2520, ChemoPharma Nigeria) and cadmium chloride (#02,416 LOBA-Chemie, India) and all other chemicals used in this study were of analytical grade.

### Experimental design

**Figure 1:**
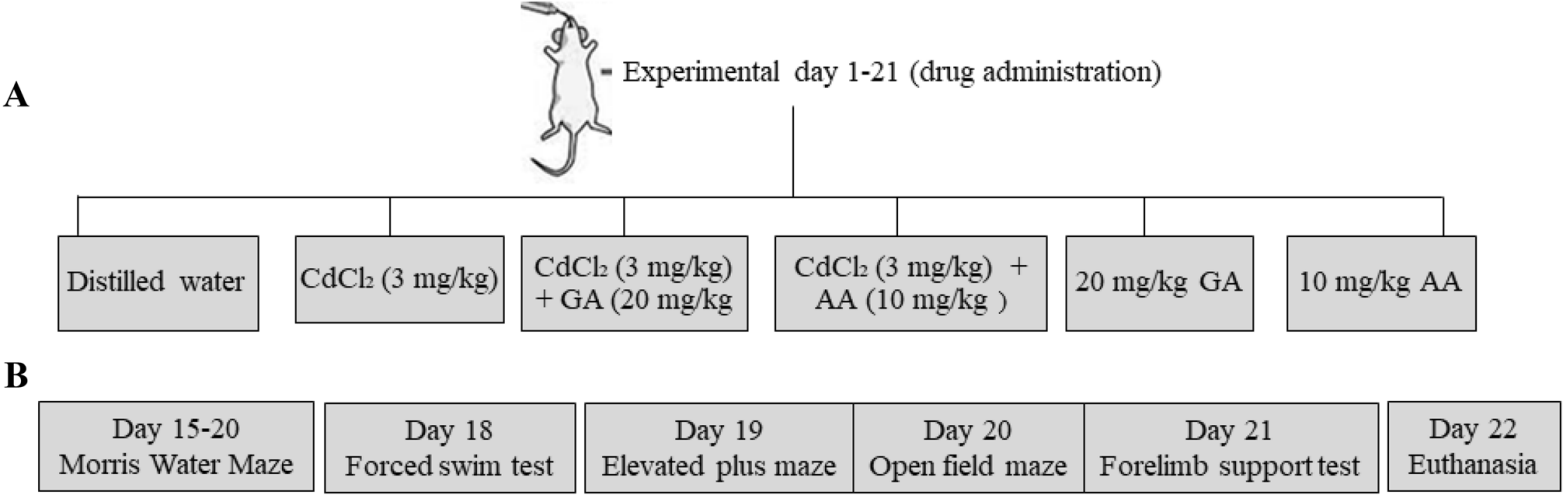
(**A**) Schematic diagram of experimental groups (**B**) Outline of behavioral assessments.

The 72 experimental rats were randomly divided into six groups (A-F; *n* = 12/group; 6 males and 6 females/group). Group A (control) received distilled water only; Group B (CdCl_2_) received 3 mg/kg of cadmium chloride; Group C (CdCl_2_ + GA) 3 mg/kg cadmium chloride and 20 mg/kg of gallic acid; Group D (CdCl_2_ + AA) 3 mg/kg cadmium chloride and 10 mg/kg ascorbic acid; Group E (GA alone) 20 mg/kg gallic acid and Group F (AA alone) 10 mg/kg body weight ascorbic acid. All chemicals were dissolved in distilled water and treatments were administered in a single daily dose via oral gavage for 21 consecutive days (Fig. 1A).

### Ethical statement

The National Institute of Health Guide for the Care and Use of Laboratory Animals (NIH Publications No. 80–23 revised, 1996) was strictly adhered to during the study. Efforts were also made to reduce pain and stress in the experimental animals. The University of Ibadan Animal Care and Use Ethics Committee approved the study (UI-ACUREC/18/0026) in line with ARRIVE guidelines.

### Daily body weight change

The body weights of the rats were measured daily throughout the study. The weight on the first day of the study was regarded as the baseline weight. We thereafter calculated the percentage of initial body weight daily from the baseline weight of the animals as:

$$ {\text{Percentage}}\,\,{\text{of}}\,\,{\text{initial}}\,\,{\text{body}}\,\,{\text{weight}}\,\,\left( \% \right)\frac{{\left( {{\text{weight}}\,\,{\text{on}}\,\,{\text{day}}\,\,n - {\text{baseline}}\,\,{\text{weight}}} \right)}}{{{\text{Baseline}}\,\,{\text{weight}}}} \times 100\% . $$

### Morris water maze (MWM) test

The maze was a rounded water pool with concealed spherical hidden escape platform placed about two inches below the water surface^23^. The water was made opaque with powdered non-fat milk. The pool was marked north, south, east and west with the escape platform fixed at a specific location. Each rat was placed into the pool from different start points randomly from the 4 locations and allowed to search for the escape stage. The time spent to locate the escape platform was recorded. If the platform was not found after 120 s, the animals were directed towards it and allowed to stay on it for at least 15 s. Individual animals underwent four tests (corresponding to the four different start locations) per day for five uninterrupted days. The animals were allowed inter-trial rest of up to one hour. This represents the acquisition phase of the test. On the 6^th^ day, a single probe test was carried out to assess spatial memory. During this phase, the escape platform was removed. The retention of the former position of the platform was measured by evaluating the period the animal spent in the target quadrant and the number of crosses within it. The time spent at the opposite of the target quadrant was also recorded and the test documented manually by two independent viewers blinded to the treatment protocol. The MWM commenced fifteen days after the onset of drug administration (Fig. 1B).

### Forced swim test (FST)

The forced swim test (FST) is one of the most commonly used tests for depressive-like behavior in rodents^24^. Rats were placed in a container filled with clean water (24 ± 1 °C) and were monitored for six minutes^25^. The onset and duration of immobility were recorded using a timer. This test was performed on experimental day 18 (Fig. 1B).

### Open field maze (OFM) test

The open field was used to assess anxiety and locomotion. The apparatus was a box measuring 72 × 72 cm × 36 cm high. One of the walls was covered with a plexiglass so the rats could be visible in the apparatus. The floor area was separated into 25 squares (20 × 20 cm), defined as 9 central and 16 peripheral squares. At the beginning of the test, each animal was placed in the center of the apparatus and then allowed to freely explore it for 5 min. The parameters recorded were line crossing which is the total number of squares visited (a measure of anxiety and locomotion), rearing (the number of times rat stood on its hind legs), grooming (number of times the animal spent licking or scratching itself while stationary), freezing (the duration with which the animal was completely stationary), center square duration (time spent in the central squares of the box), Stretched-Attend Posture (frequency with which the animal displayed forward elongation of the head and the shoulders followed by retraction to the original position) and defecation (the number of fecal boli). The OFM test was performed twenty (20) days after the onset of drug administration.

### The elevated plus maze test

The elevated plus maze test was made of two open arms (50 × 10 cm) crossed at right angles with two opposing closed arms of equal sizes. Each animal was placed at the center of the maze, facing one of the closed arms, and allowed to freely explore the arena for 5 min. The anxiety-related measures taken include the frequency and duration with which the animals visited the open arms and the closed arms.

### Forelimb support (hanging wire test)

A 2 mm thick metallic wire was tightly attached to a frame to avoid vibration or unwanted displacement of the wire during the test. Individual rat was placed on the 2 mm diameter hanging wire with its forelimb and monitored for a period of 2 min. The period of time it took the animal to stay on the wire before falling was recorded. Rats were tested twice and allowed about 2 h of rest in between. The hanging wire was performed twenty-one days after the commencement of drug administration.

In between testing, all the apparatus were cleaned with 70% ethyl alcohol to eliminate odor cues of previously tested rat. All behavioral tests were carried out by an experimenter blinded to the treatment groups.

### Animal sacrifice

Twenty-four hours after the behavioral tests, each rat was deeply anesthetized with sodium pentobarbital (10 mg/100 g body weight) and transcardially perfused with physiological saline, followed by 4% paraformaldehyde in 0.1 M phosphate buffer (PB, pH 7.4) at 4 °C. Brains were removed and placed in 10% formaldehyde and 30% NBF solution for histopathology and immunohistochemistry respectively.

### Haematoxylin and Eosin Staining

The brains were processed routinely for paraffin embedding. 5 μm sections were obtained with rotary microtome and processed for Haematoxylin and Eosin staining as previously documented^26^. Sections were observed with light microscope and photomicrograph taken with a Leica LAS-EZ Microscope installed with Leica software application suite version 3.4.

### Luxol fast blue (LFB) staining

Brain sections (5 μm thick) were deparaffinized in xylene and thereafter hydrated to 5% ethanol. The sections were then incubated in LFB (#26681-01, Electron Microscopy Sciences, USA) solution (0.1% LFB in 95% ethanol, 5% methanol with 0.05% glacial acetic acid) at 56 °C for 16 h. Thereafter, the sections were differentiated with 0.05% lithium carbonate solution (#26681-04, Electron Microscopy Sciences, USA) for 30 s followed by 70% ethanol. The sections were further differentiated in 95% ethanol and rehydrated before mounting with Eukitt^®^ Quick-hardening mounting medium (#03989, Sigma, USA).

### Immunofluorescence

Immunofluorescence was performed by de-paraffinizing the brain sections in xylene and rehydration in graded percentages of alcohol. Antigen retrieval was done by placing the slides in 10 mM citrate buffer at 95–100 °C for 15 min. The slides were thereafter blocked in blocking buffer (2X casein/10% goat serum/0.1% Triton X-100 pH 7.4) and later incubated with the following primary antibodies: anti-NeuN polyclonal antibody (1:500; #MAB377, Millipore^®^), anti-MBP rabbit polyclonal antibody (1:500; #PA5-78397, Invitrogen^®^). The sections were rinsed 3 times (5 min each) in PBS and then incubated with secondary antibodies conjugated with Alexa dyes (AF-488, #A11001, Invitrogen and AF-647, #A21245, Life Technologies) at room temperature for 1 h. Sections were then washed 3 times (5 min each) followed by the addition of 2 drops of mounting media with DAPI (Invitrogen #P36935) before sections were cover slipped. The pixel intensities for MBP and NeuN images were analyzed using ImageJ software.

### Statistical analysis

Data were analyzed using a one-way analysis of variance (ANOVA) followed by Tukey’s post hoc test for comparison between groups. All tests were used with significance set at *p* < 0.05. The data were presented as mean ± standard deviation.

Data analysis indicated no sex differences in any of the parameters investigated above hence we combined data from males and females experimental rats.

## Results

### CdCl_2_ administration led to reduction in body weight

**Figure 2:**
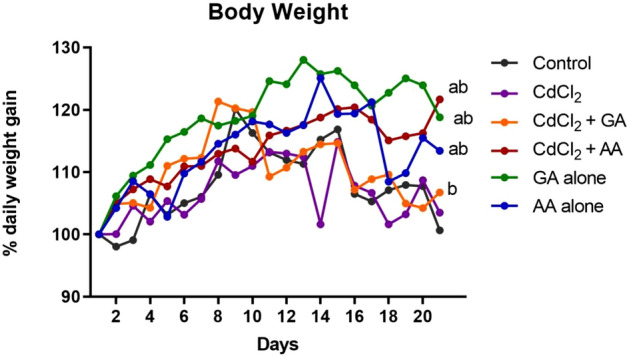
The body weight gain of rats expressed as percent of initial body weight exposed to Cadmium (CdCl_2_) only, CdCl_2_ and gallic acid (GA), CdCl_2_ and ascorbic acid (AA), GA alone and AA alone compared with the control (*n* = 12/group).

We observed a gradual increase in body weight across all the groups in the first nine days of this study. However, by day 10, 11 and 14 there was a decline in body weight gain in the CdCl_2_ + AA, CdCl_2_ + GA and CdCl_2_ groups respectively. The decrease in body weight gain in these groups was initially not significant but by day 16 till the end of the experiment this decrease in weight had become statistically significant relative to the GA alone and AA alone groups (Fig. 2).

### Gallic acid improved the learning pattern of rats following impairment by CdCl_2_

**Figure 3:**
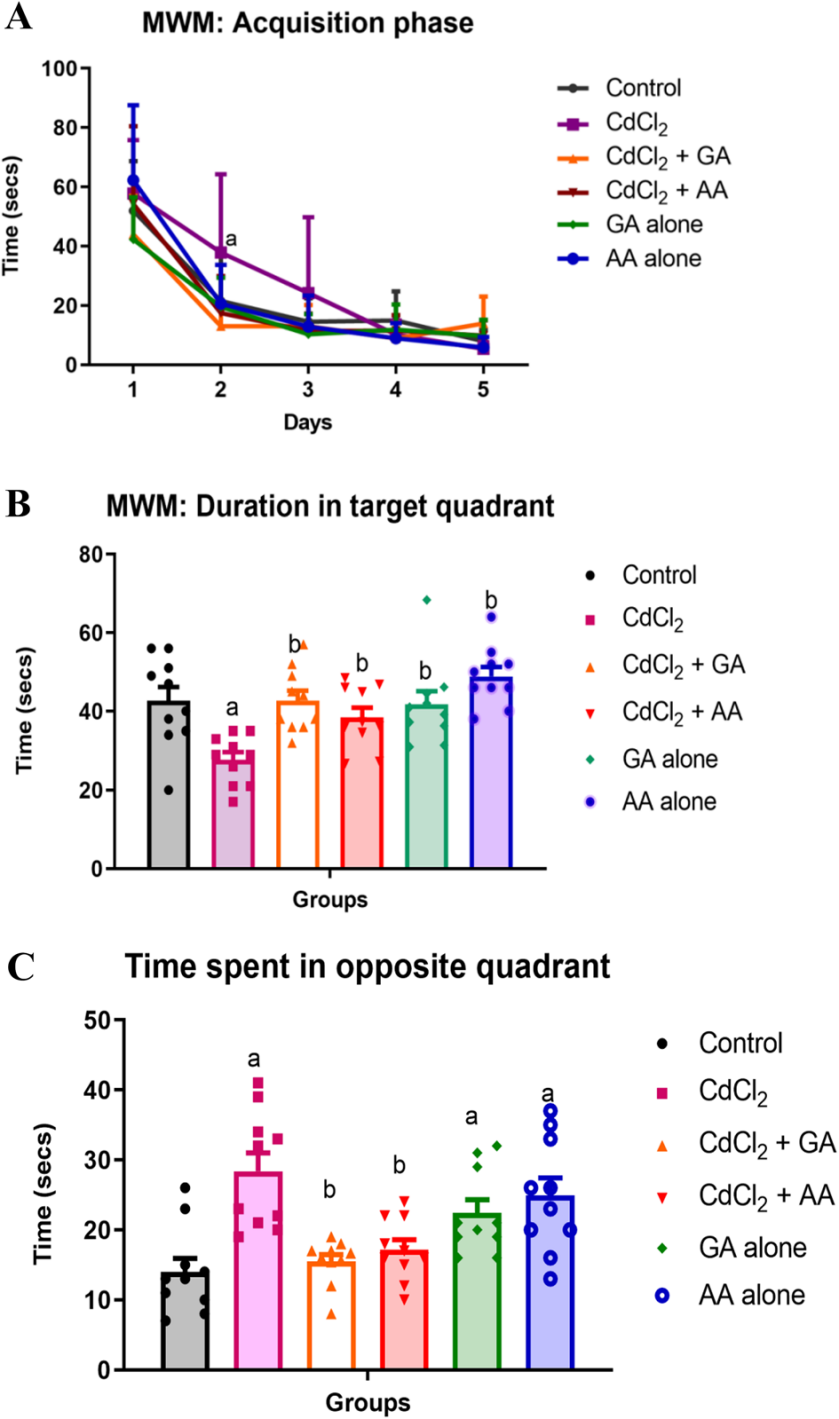
(**A**) Learning curve during the acquisition phase of the Morris water maze test (**B**) The probe trial of the Morris water maze showing time spent in the target quadrant and (**C**) The time spent in the opposite quadrant. Values are presented as mean ± Standard Deviation *n* = 12/group; ^a^indicates significant difference at *p* < 0.05 compared with control; ^b^Indicates significant difference at *p* < 0.05 compared with CdCl_2_(one-way ANOVA; *n* = 12). *GA* gallic acid, *AA* ascorbic acid.

During the acquisition trial of the Morris water maze we assessed the learning of the rats as the time it took the rat to find and climb onto the escape platform (escape latency). In all the experimental groups, the escape latency diminished over the 5-day training period (Fig. 3A). Repeated-measures one-way ANOVA showed a significant reduction in escape latency with training (F (6.031, 63.63) = 2.514) *p* = 0.0316. On the 6th day when the escape platform had been removed we observed the CdCl_2_ group had a significant decrease in time spent at the target quadrant when compared to the other groups. Remarkably, the time spent in the target quadrant by the CdCl_2_ + GA group was similar to control, which was significantly (*p* = 0.029) increased when compared to the other groups (Fig. 3B). On the contrary, the CdCl_2_ group spent a significantly (*p* = 0.0001) increased time in the opposite quadrant while the control and the CdCl_2_ + GA group had similar significantly (*p* = 0.041) reduced duration in the opposite quadrant (Fig. 3C).

### The depressive-like behavior exhibited in the CdCl_2_ group was ameliorated following concurrent administration of gallic acid

**Figure 4:**
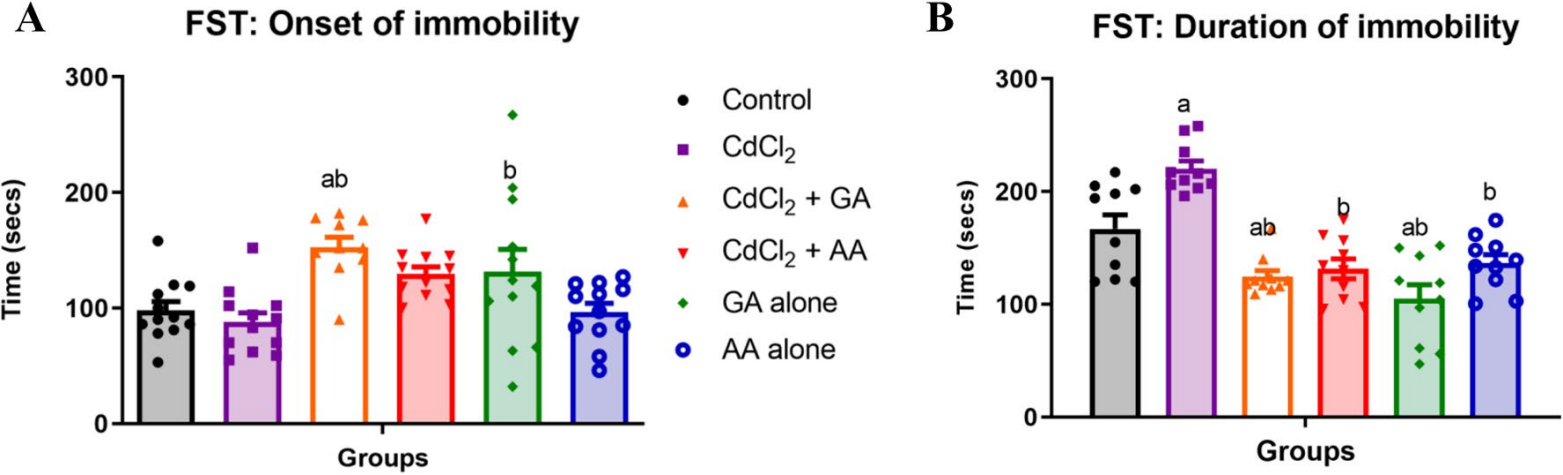
The effects of Gallic acid (GA) and ascorbic acid (AA) on CdCl_2_-induced depression. (**A**) is the onset of immobility. (**B**) is the duration of immobility. Values are presented as mean ± SD *n* = 12/group. ^a^significant difference at *p* < 0.05 versus control, ^b^significant difference at *p* < 0.05 versus CdCl_2_.

During FST, we observed that the onset of immobility was significantly higher in the CdCl_2_ + GA and GA alone groups relative to other groups. Similarly, the duration of immobility was significantly increased in the GA alone group, while the CdCl_2_ + GA and CdCl_2_ + AA groups had significantly lower duration of immobility relative to GA alone and control groups. Overall, our results showed that the concurrent administration of GA or AA with CdCl_2_increased struggling behavior in the experimental rats (Fig. 4).

**Figure 5:**
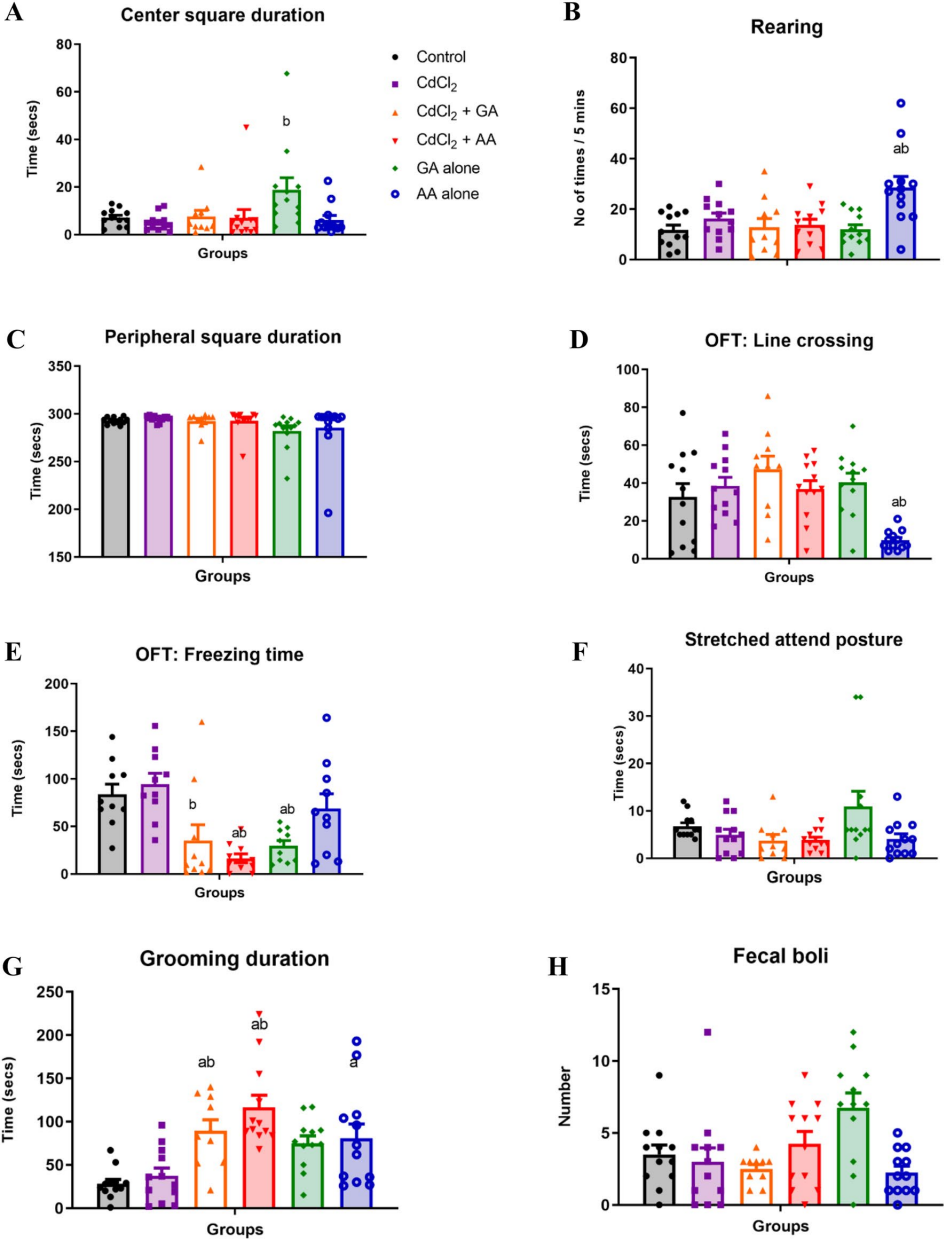
The effects of gallic acid and ascorbic acid on CdCl_2_-induced anxiety and locomotion in the open field test showing (**A**) freezing time (**B**) line crossings (**C**) rearing frequency(**D**) grooming duration (**E**) duration of stretched attend posture (**F**) center square duration (**G**) peripheral square duration and (**H**) number of fecal boli. Values are presented as mean + SD. ^a^indicates significant difference at *p* < 0.05 compared with control ^b^indicates significant difference at *p* < 0.05 compared with CdCl_2_ only group. *GA* gallic acid, *AA* ascorbic acid.

Since we observed that CdCl_2_ + GA and CdCl_2_ + AA groups had increased escape or struggling-directed behavior (as depicted by significant decreased duration of immobility) compared to the CdCl_2_ alone group we subsequently sought to explore their locomotor activity since most antidepressants are known to reduce locomotion. We consequently investigated their actions on locomotion using the open field maze (OFM). We used the OFM as a positive control to further analyze the effect seen in the FST. Our results indicated similarity in line crossing across all the groups except in the AA only group where number of lines crossed was significantly reduced. Interestingly, we also observed a significant reduction in freezing time in the CdCl_2_ + GA and CdCl_2_ + AA groups relative to the CdCl_2_ alone group. In addition, stretched attend posture, number of fecal boli, peripheral square and grooming duration were similar across all the experimental groups (Fig. 5).

**Figure 6:**
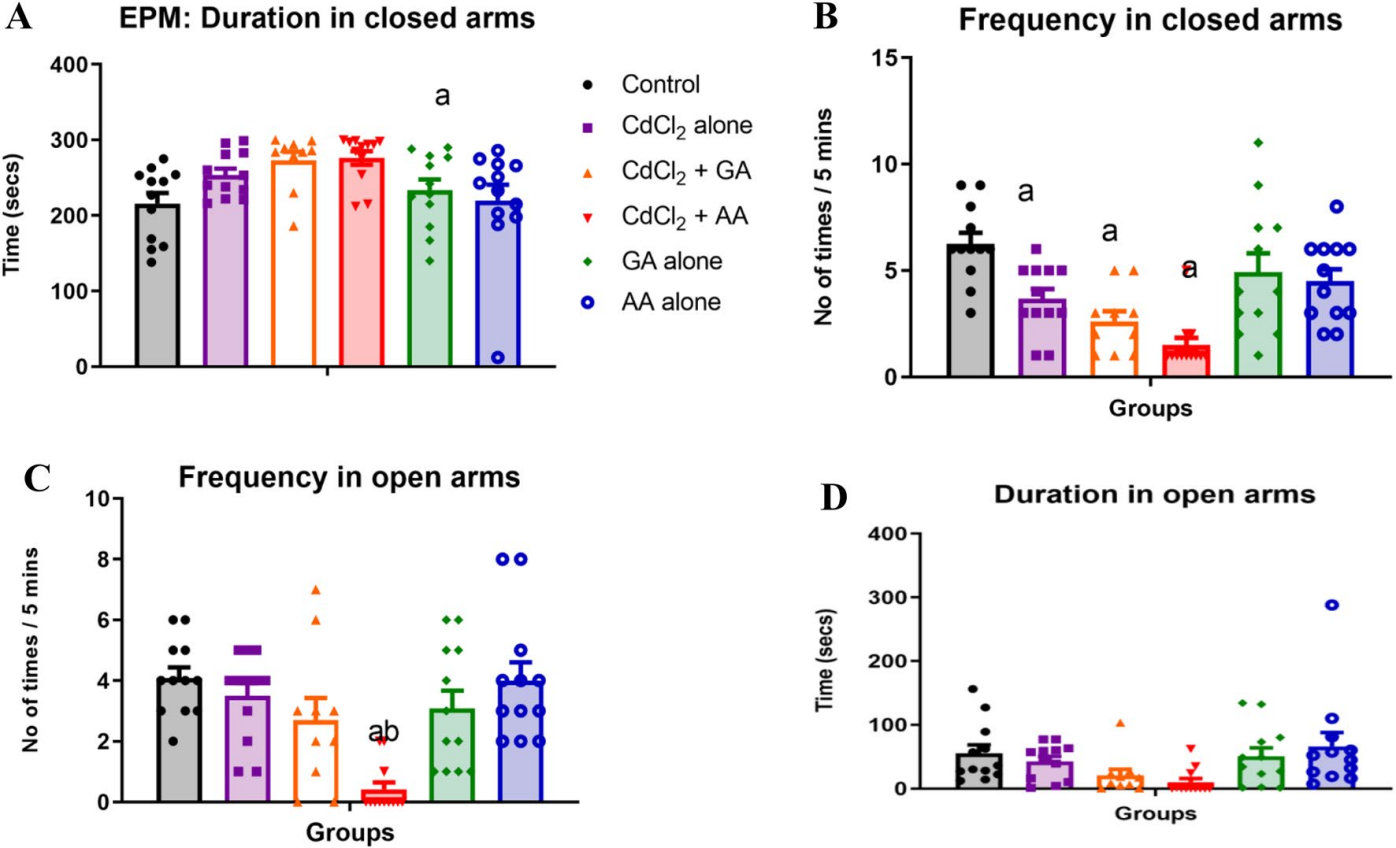
The effects of gallic and ascorbic acids on CdCl_2_-induced anxiety in the elevated plus maze test. (**A**) Number of entries into the open arms (**B**) time spent in the open arms (**C**) number of entries into the closed arms and (**D**) time spent in the closed arms. *n* = 12/group, Values are presented as mean + SEM. ^a^indicates significant difference at *p* < 0.05 compared with control ^b^indicates significant difference at p < 0.05 compared with CdCl_2_ only group. *GA* gallic acid, *AA* ascorbic acid.

In the elevated plus Maze test, One-way analysis of variance (ANOVA) revealed a significant decrease in the frequency of entry into the open arms in the CdCl_2_ + AA group when compared with the control (*p* = 0.0014) and CdCl_2_ alone (*p* = 0.0006) groups. In addition, we observed a significant decrease in the frequency of entry into the close arms in all groups administered CdCl_2_, whether alone or concurrently with GA or AA. However, the time spent in the open and closed arms of the maze were similar across all the groups (Fig. 6).

### CdCl_2_ did not alter hanging latency on the forelimb suspension test

**Figure 7:**
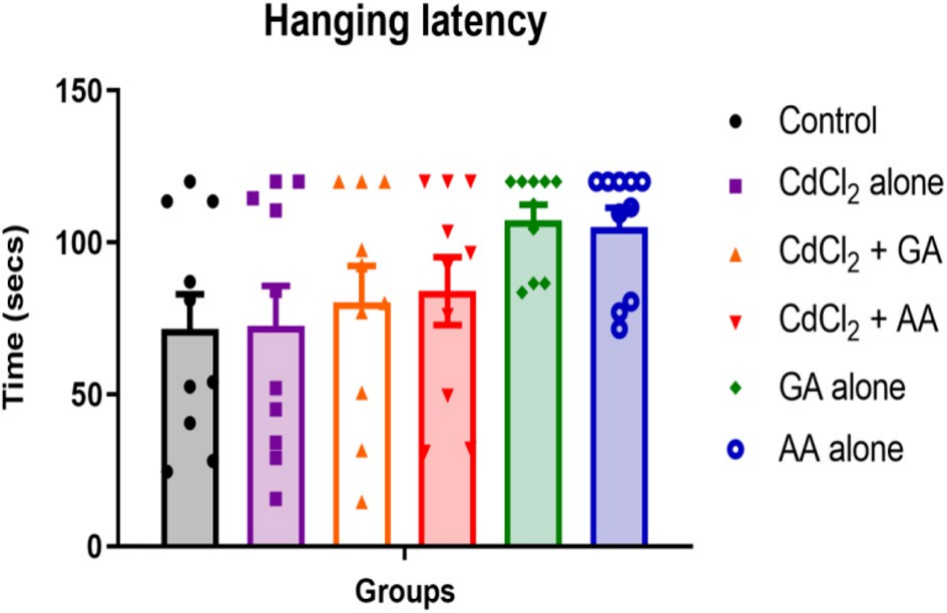
The hanging latency in experimental rats following administration of gallic acid, ascorbic acid and CdCl_2_ Values are presented as mean + SEM (*n* = 12/group).

The mean hanging latency was 71.45 ± 11.52, 72.45 ± 13.19, 80.35 ± 11.92, 84.05 ± 11.18, 107.4 ± 5.026 and 105.0 ± 6.407 in the control, CdCl_2_ alone, CdCl_2_ + GA, CdCl_2_ + AA, GA alone and AA alone groups respectively. Our results indicate similarity in mean hanging latency across all groups (Fig. 7).

### Gallic acid rescued myelin disruption and reduced astrocytic activation following CdCl_2-_induced neurotoxicity

**Figure 8:**
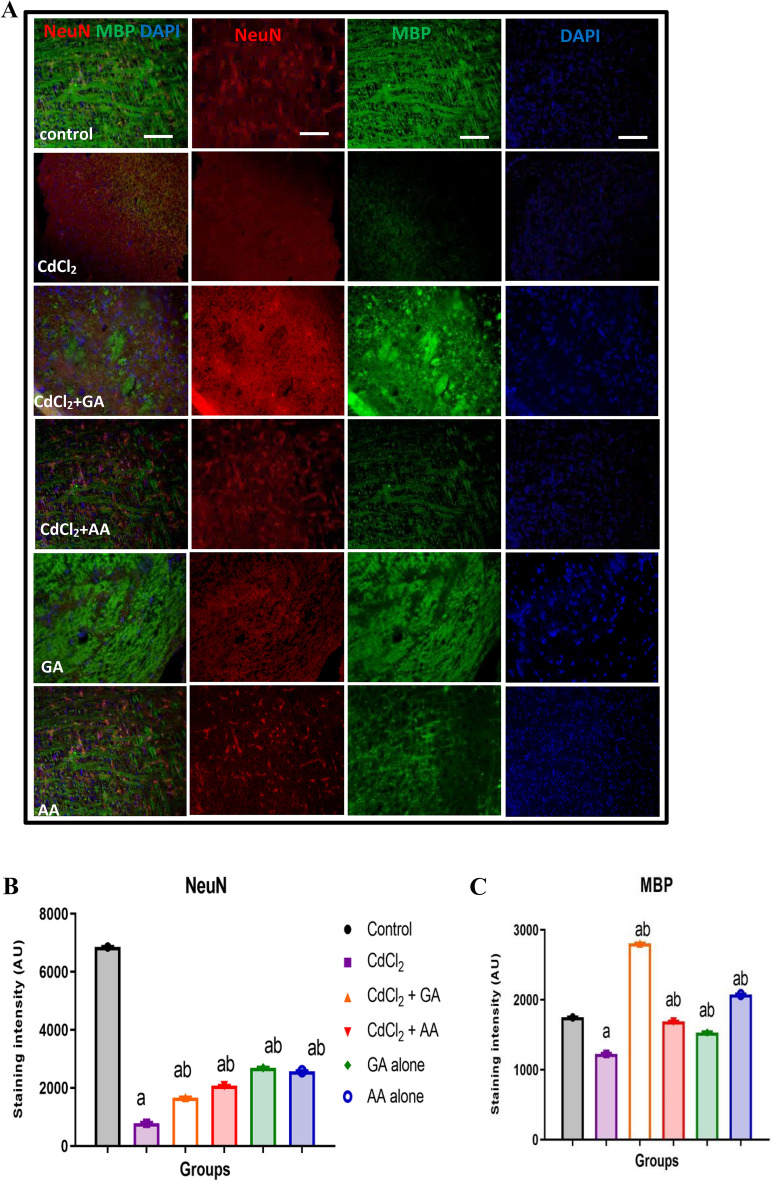
(**A**) Representative double immunofluorescence stainingphotomicrograph showing neuronal nulei (NeuN; red), myelin basic protein (MBP; green) and cell nuclei (DAPI; blue) in the brain cortex in experimental rats. Quantification ofstaining intensity of (**B**) NeuN and (**C**) MBP was performed using ImageJ software. *GA* gallic acid, *AA* ascorbic acid.

**Figure 9:**
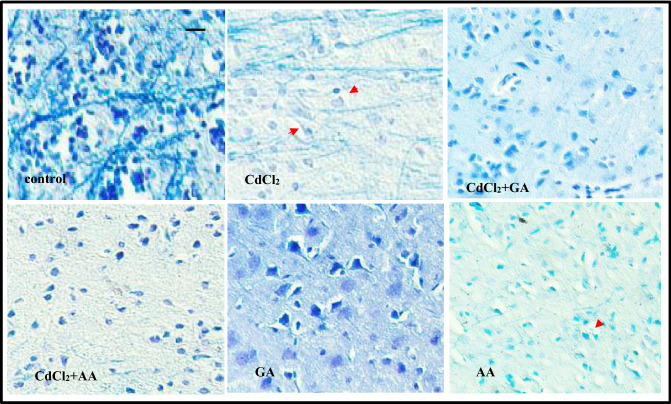
Representative images of Luxol fast blue staining of subcortical white matter of experimental rat showing the myelin density of the tract. Red arrowheads indicate vacuolation within the white matter. Note: the pale staining intensity in the CdCl_2_ and AA groups compared to other groups. *GA* gallic acid, *AA* ascorbic acid (scale bar = 50 µm).

**Figure 10:**
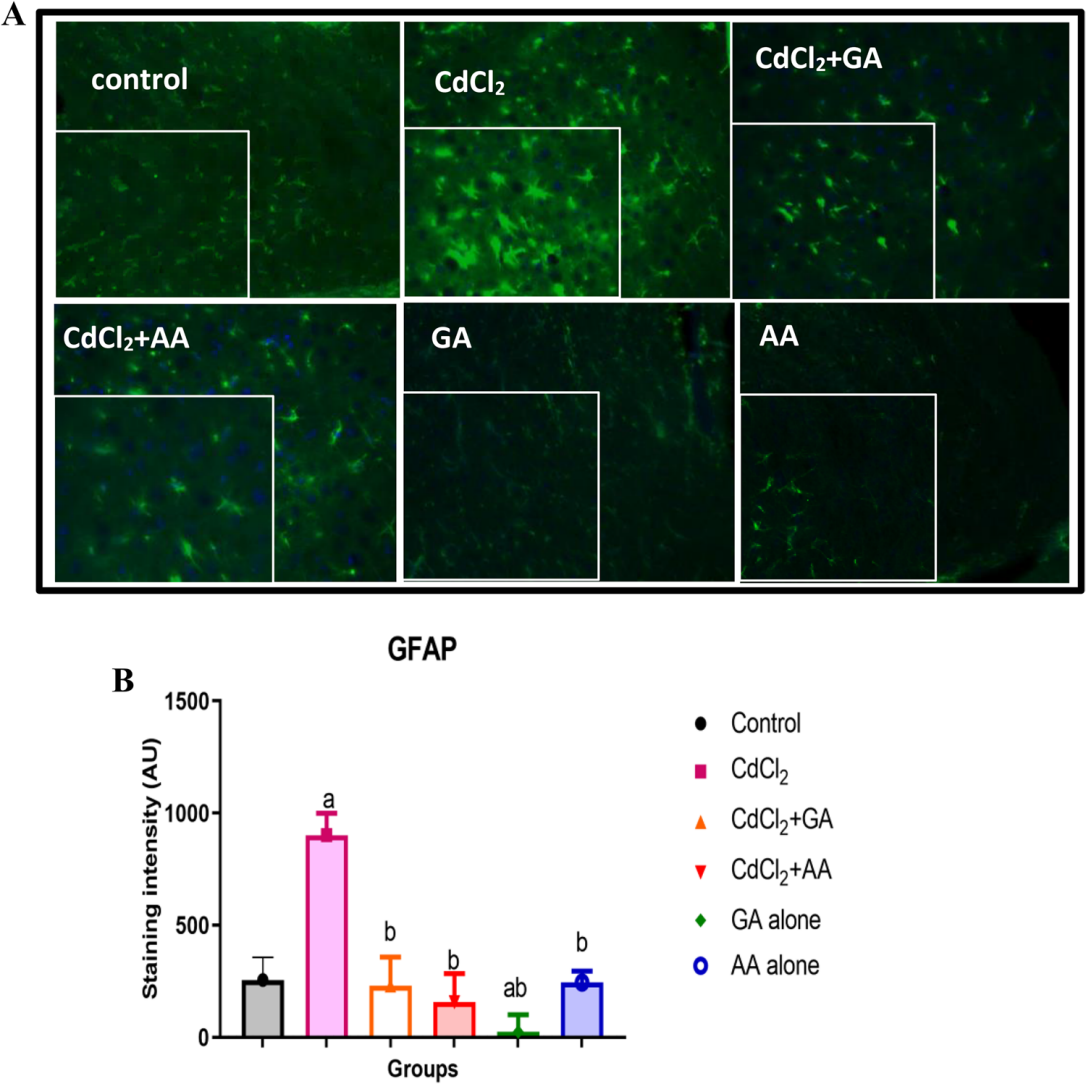
(**A**) Immunofluorescence showing Glial fibrillary astrocytic protein (GFAP) positive cells following the administration of CdCl_2_, GA and AA. (**B**) Quantification of mean astrocyte count. *GA* gallic acid, *AA* ascorbic acid scale bar = 50 µm).

We further investigated the effects of CdCl_2_ on integrity of myelin sheaths using anti-MBP antibodies and Luxol Fast Blue (LFB) staining. Anti-MBP immunofluorescence staining showed a reduction in staining intensity in the cortex of CdCl_2_ and CdCl_2_ + AA rats (Fig. 8A). We observed a significant decrease in expression of neuronal nuclei (NeuN) protein across all groups relative to control (Fig. 8B).Quantitative examination of staining intensity indicated a significant reduction (*p* < 0.05) in the CdCl_2_ and CdCl2 + AA rats compared with control and CdCl_2_ + GA rats (Fig. 8C). Furthermore, we observed that LFB revealed the white matter tracts appeared thinner with vacuolations and stained pale in rats administered with CdCl_2_ (arrows, Fig. 9). Conversely, deeply stained myelinated fibers were detected traversing through the white matter in control and CdCl_2_ + GA groups (Fig. 9). Similarly, the CdCl_2_ group had less intensely stained, disorganized cerebellar white matter with significant decrease in number of positive MBP cells. Likewise, our results showed that cells positive for GFAP appear to be enlarged and activated in the CdCl_2_ rats (Fig. 10A). In addition, quantification of the expression of GFAP revealed a significant elevation in the CdCl_2_ group relative to the other test groups (Fig. 10B).

### CdCl_2_-induced Purkinje cell degeneration was ameliorated by gallic and ascorbic acids

**Figure 11:**
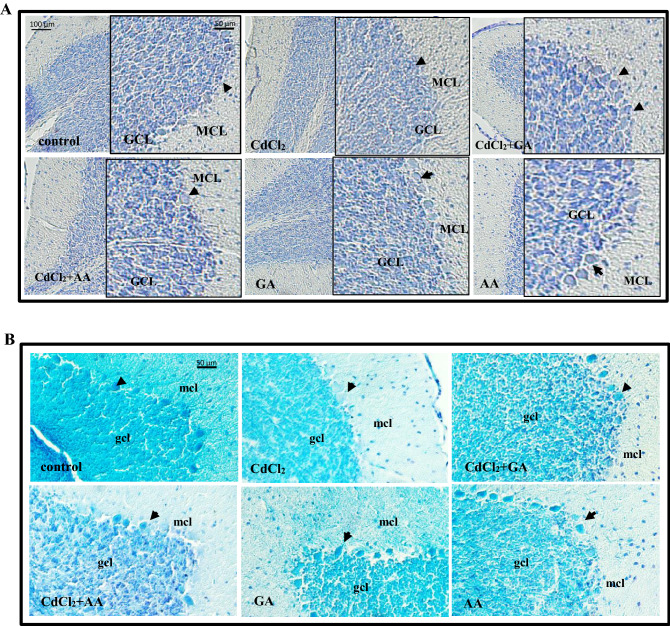
The cerebellum of experimental rats showing the molecular, Purkinje and granule cell layers with (**A**) H&E staining and (**B**) Luxol fast blue stain. Control, GA and AA groups had organized molecular cell layer with discrete stellate and basket cells. Large pyriform-shaped cells were also observed in the Purkinje cell layer with small deeply stained granular cell layer. Note the disorganization in the cells of the Purkinje layer in the CdCl_2_ group. Molecular cell layer (MCL), Purkinje cell (black arrowheads) and granular cell layers (GCL). *GA* gallic acid, *AA* ascorbic acid.

In Wistar rats, the cerebellum is made up of molecular, Purkinje and granular cell layers (Fig. 11). Our results showed a disruption with lack of clear organization of the Purkinje cell layer in the CdCl_2_ group. Additionally, the remaining Purkinje cells appeared disorganized with distorted cytoplasms. We further investigated this apparent neuronal loss, by carrying out Purkinje cell counts in the different treatment groups. Our results revealed a significant decrease in entire Purkinje cell number in the CdCl_2_ group (9% decrease in total number; *p* = 0.032). However, the total number of Purkinje neurons were similar in the control and groups administered gallic acid.

## Discussion

Cadmium has been reported by several studies to induce behavioral deficits such as memory impairment, anxiety and depression^27–29^. It does this primarily through the generation of reactive oxygen species (ROS)^9^. Gallic acid is a phenolic acid with antioxidant activity which has been reported over the past years to be effective against nervous system disorders such as depression, anxiety, ischaemia, Alzheimer’s and Parkinson’s diseases^14^. Oral supplementation with ascorbic acid has also been used to mitigate heavy metal toxicity as well as protect against cadmium induced toxicity^18^. The present study was carried out to evaluate the effects of gallic acid on cadmium-induced neurotoxicity with focus on neuro-behavioral deficits such as memory loss, anxiety, depression and loss of motor coordination; and compare its effects with that of ascorbic acid, a common antioxidant.

The decrease in body weight in the CdCl_2_ group reported in this study is in consonance with the reports of El-Demerdash et al.^30^ and Prabu et al.^31^. This observed weight loss may be as a result of the association of CdCl_2_ administration with disruption in glucose regulation resulting in impaired protein, carbohydrate and lipid metabolism as previously highlighted^32^. Conversely, Young et al.^33^ reported an increase in body weight following CdCl_2_ administration. This variability in results may be due to the different exposure routes and dosage of CdCl_2_ in the studies. While in the previous study, CdCl_2_ was administered in water, our study utilized oral gavage to ensure that the full dose of CdCl_2_ was delivered directly to the stomach. However, the association of CdCl_2_ administration with weight alteration is outside the aim of this study.

The Morris water maze in rodents is a test for spatial learning and memory that relies on external or extra-maze cues to locate a submerged escape platform^34^. It tests for hippocampal dependent learning, which includes acquisition and long-term memory and plays an important role in the validation of cognitive studies in rodent models^35^. In the present study, learning occurred across all the groups but there was a significant decrease in time to locate the escape platform in the CdCl_2_ alone group when compared with the control which indicates that CdCl_2_ caused a learning deficit. In the probe phase, CdCl_2_ alone group showed a significant reduction in the time spent in the target quadrant when compared with the other experimental groups which is indicative of an impairment in spatial memory. It is anticipated that rats would use the visual stimuli from the cues within the testing room to form a “spatial orientation map” in their brain leading to a decrease in escape latency over the course of the test. However, this was not so in the CdCl_2_ only, this corroborates the findings of Halder et al.^28^ that cadmium causes a decrease in retention memory. Remarkably, CdCl_2_ rats treated with gallic and ascorbic acid spent similar time at the target quadrant as the control group in the probe trial of the MWM test, indicating a recovery in memory retention. Although CdCl_2_ caused a significant impairment in memory retention, spatial learning was still evident during the acquisition phase of the test as the animals spent reduced time in finding the escape platform with increasing number of trials. Interestingly, a study by Wang et al.^36^ aligns with this finding that cadmium-treated mice exhibit a deficit in this spatial memory and did not show improvements even after nine weeks of the cessation of cadmium.

Increased cadmium levels have also been linked to the presence of depressive-like behavior^29^. The forced swim test is a widely used test to evaluate depressive disorders^24^. The increase in the duration of immobility that is seen in the cadmium alone group is indicative of anxiety as earlier documented^25,37^. Remarkably, gallic acid and ascorbic acid treated groups reduced significantly the duration of immobility indicating their anti-depressive like potential in cadmium-induced toxicity.

The fear-related behaviors ranging from locomotion, freezing duration, exploratory activity and emotional state examined in our study revealed a dramatic decline in anxiety-like behaviors following administration of gallic and ascorbic acids. This outcome further supports that ascorbic acid promotes anxiolytic effects in diverse animal models as previously documented in zebrafish^38^, mice^39^ and rats^40^. Although, the exact mechanisms of anxiolytic activity of these compounds remains to be decisively established, reports have suggested anti-oxidant related decrease of brain oxidative stress and NMDA antagonism^41,42^. On the other hand, some authors have associated this activity to their ability in reducing circulating levels of cortisol, a stress hormone^43^.

The hanging wire test is a test of muscle strength^44^. Cadmium has been previously linked with motor activity impairment^45^. Our study nevertheless showed no significant difference in hanging latency across all the groups. This incongruence in hanging latency across the groups in this study may be due to the shorter duration of administration of the metal in our study compared to earlier reports^14,45^ that had a 28-day of administration of cadmium.

Demyelination is due to several pathologies in the brain such as inflammation, viral infection, focal compression, metabolic derangements and ischaemia^46^. Accumulation of cadmium has been linked to loss of myelin and reduced expression of myelin basic protein (MBP) in the cerebral and cerebellar cortices of rats^47^. Our study showed a reduced myelin expression in the cadmium treated group which was restored in both the ascorbic and gallic acid treated groups. The loss of cerebral neurons observed in LFB and demyelination in both MBP and LFB may be responsible for the impaired memory that was observed in the Morris water maze test. This study suggests that the demyelination could be due to an inflammatory process.

Astrocytes are cells that produce intermediate filaments such as glial fibrillary acidic protein (GFAP). Astrocytic activation (or astrocytosis) results from almost all insults or injury to the brain. The increased GFAP expression could also indicate an ongoing inflammation in cadmium toxicity leading to astrogliosis^48^. This supports the claim of astrocytic involvement in heavy metal neurotoxicity^49^. This astrogliosis was however reversed in the groups treated with gallic acid and ascorbic acid. Plant metabolites such as soybean supplementation in diet has also been shown to reduce arterial and cardiac injuries induced by cadmium^50^. Hence we propose that gallic acid being a secondary polyphenol with antioxidant potential could be beneficial against damages caused by cadmium chloride-induced experimental neurotoxicity and behavioral alterations. These effects can both be linked to their antioxidant activities.

## Conclusion

Cadmium chloride caused a deficit in learning and memory with accompanying evidences of anxiety and depressive-like behavior which are indicative of neurotoxicity. Concurrent administration of gallic acid and ascorbic acid ameliorate the neurotoxic effects induced by cadmium. Although gallic acid showed a better neuroprotective effects over ascorbic acid; these compounds should be explored in population that are environmentally or occupationally exposed to toxic levels of cadmium.

## Data Availability

The supporting data of this study are available from the corresponding author upon reasonable request.

## Abbreviations

EPM: Elevated plus maze
GFAP: Glial fibrillary acidic protein
MBP: Myelin basic protein
MWM: Morris water maze
NeuN: Neuronal nuclei
